# Multi-Trait Genomic Prediction Improves Accuracy of Selection among Doubled Haploid Lines in Maize

**DOI:** 10.3390/ijms232314558

**Published:** 2022-11-22

**Authors:** Haixiao Hu, Yujie Meng, Wenxin Liu, Shaojiang Chen, Daniel E. Runcie

**Affiliations:** 1 Department of Plant Sciences, University of California, Davis, CA 95616, USA; 2 National Maize Improvement Center of China, College of Agronomy and Biotechnology, China Agricultural University, Beijing 100193, China; 3 Department of Plant Biology, University of California, Davis, CA 95616, USA

**Keywords:** multi-trait model, genomic prediction, doubled haploid line, haploid line, genetic correlation, DH-line-based hybrid breeding

## Abstract

Recent advances in maize doubled haploid (DH) technology have enabled the development of large numbers of DH lines quickly and efficiently. However, testing all possible hybrid crosses among DH lines is a challenge. Phenotyping haploid progenitors created during the DH process could accelerate the selection of DH lines. Based on phenotypic and genotypic data of a DH population and its corresponding haploids, we compared phenotypes and estimated genetic correlations between the two populations, compared genomic prediction accuracy of multi-trait models against conventional univariate models within the DH population, and evaluated whether incorporating phenotypic data from haploid lines into a multi-trait model could better predict performance of DH lines. We found significant phenotypic differences between DH and haploid lines for nearly all traits; however, their genetic correlations between populations were moderate to strong. Furthermore, a multi-trait model taking into account genetic correlations between traits in the single-environment trial or genetic covariances in multi-environment trials can significantly increase genomic prediction accuracy. However, integrating information of haploid lines did not further improve our prediction. Our findings highlight the superiority of multi-trait models in predicting performance of DH lines in maize breeding, but do not support the routine phenotyping and selection on haploid progenitors of DH lines.

## 1. Introduction

The doubled haploid (DH) technology based on in vivo haploid induction has become one of the most important tools in maize breeding during the past two to three decades [1], and it has been widely adapted in public and private maize breeding programs all over the world [2]. The technology samples the segregating gametes of source germplasm, usually a biparental cross, and produces completely homozygous lines within two growth seasons [3]. This greatly accelerates line development and reduces its cost, relative to the conventional method of recurrent selfing of segregating materials for six to eight generations to reach the desired level of homozygosity.

A typical DH-line-based hybrid breeding scheme includes a first stage of selection among the DH lines per se followed by one or several stages of testcross hybrid selection [4]. Although the DH technology enables fast and efficient development of pure lines, generating large numbers of DH lines from two opposite heterotic pools results in a tremendous number of possible hybrid combination crosses that need to be tested in multi-environment field trials. This imposes great challenges in field testing with limited budgets and therefore may counterbalance its potential in accelerating hybrid development. Genomic prediction using genome-wide DNA-based molecular markers can be used to predict the field performance of DH lines per se or in hybrids without a need to grow them all in the field. This enables plant breeders to pick a subset of the best candidate, target a limited number of the best potential crosses to make, and finally evaluate them in multi-environment field trials. In addition, since each DH line is derived from a haploid plant whose chromosomes can be doubled spontaneously or by applying chemical treatment such as colchicine, the selection of DH lines could be conducted when they are still haploids in the previous generation, further speeding up breeding cycles. Geiger et al. [4] found moderate to strong performance correlations between haploid and DH lines and that preselection at the haploid level could potentially result in improved per se performance at the DH level or at the hybrid cross level [4,5].

Empirical studies for predicting field performances of DH lines using genomic prediction have been reported in maize [6,7,8,9]. These studies demonstrated the usefulness of genomic prediction for preselection of DH lines in the early stage of a breeding pipeline. However, most studies used a relatively small number of yield-related or agronomic traits and predicted each trait separately using conventional univariate models. This neglects any genetic correlations between traits or between haploid and DH lines (although Wang et al. [8] also modeled the genotype by environment interaction, which is a special case of the multi-trait model). The genetic correlation is a measure of association between breeding values of individuals for a pair of quantitative traits [10]. The observed phenotypic covariance between a pair of quantitative traits can be partitioned into genetic and environmental components. Thus, the genetic correlation provides a genetic basis for the multi-trait genomic prediction. Breeders commonly collect phenotypic data on many traits and in multiple environments. Multi-trait genomic prediction models can take into account (1) the genetic correlation between traits, which enables prediction of traits that are difficult to measure directly; (2) genetic correlations of the same trait across environments, which can increase efficiency in multi-environment trials; and (3) genetic correlations between DH and haploid lines, which can borrow information from the haploid level to predict DH lines.

In this study, we analyzed phenotypic and genotypic data of a DH population derived from a biparental cross, and phenotypic data of haploid lines created from the DH lines. In both populations, a total of 35 traits, including 17 agronomic traits and 18 stalk quality traits, were evaluated in four environments. Our objectives were to (1) characterize phenotypes of the 35 traits in the DH and corresponding haploid populations; (2) estimate phenotypic and genotypic correlations between the DH and haploid lines; (3) compare genomic prediction accuracies of multi-trait models using agronomic traits to predict stalk quality traits in a single-environment trial or across multi-environment trials against conventional univariate models; and (4) evaluate whether including information of haploid lines for predicting DH lines could further improve the prediction accuracy over using DH lines alone.

## 2. Results

### 2.1. Characterizing Phenotypes of 35 Traits in DH and Corresponding Haploid Populations

In both the DH and haploid populations (Figure S1), a total of 35 phenotypic traits were evaluated in four environments: BJ2013, SJZ2013, BJ2014, and SJZ2014 (see Methods). These include nine stalk quality traits (contents of acid detergent fiber (ADF), ash (ASH), cellulose, crude protein (CP), crude fat (FAT), in vitro dry matter digestibility (IVDMD), lignin, neutral detergent fiber (NDF), and water soluble carbohydrate (WSC)), nine agronomic traits (rind penetrometer resistance measured at the middle of the internode (RPR), fresh weight of the internode (FreshWeight), dry weight of the internode (DryWeight), internode diameter, and internode length) measured from both the fourth internode above ground (FI) and the internode under the primary ear (EI), and seven agronomic traits (dry weight, ear height, fresh weight, leaf angle, leaf length, leaf width, and plant height) evaluated from the whole plant (WP) (Table S1).

**Figure 1:**
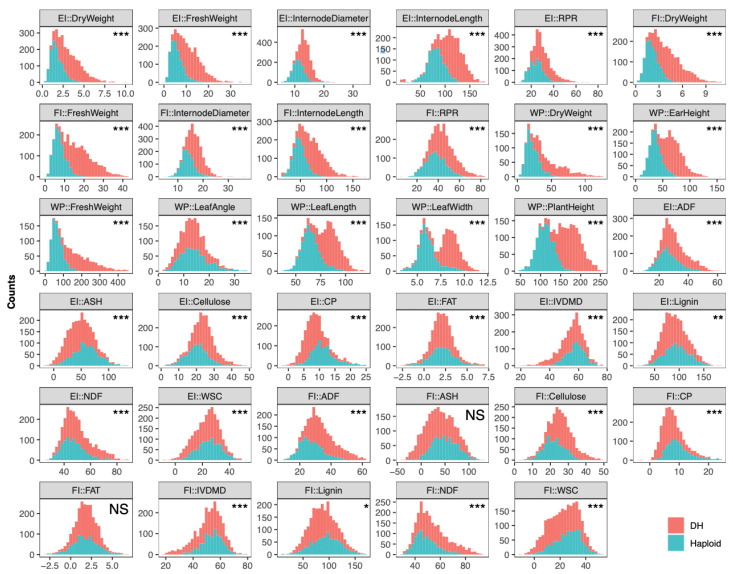
Phenotypic value distributions of the DH and haploid populations for each of the 35 traits. For each trait, histograms of the two populations were plotted independently and based on all phenotypic values across four environments. The *t*-test was applied to test the mean difference between the two populations for each trait, for which *** = significant at *p* < 0.001, ** = significant at *p* < 0.01, * = significant at *p* < 0.05, and NS = not significant.

We observed significant differences between means of the DH and haploid populations for all traits except the content of ash (FI::ASH) and crude fat (FI::FAT) measured from the fourth internode above the ground (Figure 1, Table S1). We further examined whether one population has greater dispersion than the other population for those traits using coefficient of variation, which measures the standard deviation normalized by the mean. We found that the haploid population showed greater dispersions for 13 of the 17 agronomic traits (*p* = 0.012, Figure S2) based on a *t*-test. The DH population showed larger dispersion for 11 of the 18 stalk quality traits; however, the mean difference was not significant (*p* = 0.11, Figure S2).

### 2.2. Phenotypic and Genetic Correlations between DH and Haploid Lines

To estimate phenotypic and genetic correlations between DH lines and their corresponding haploids, we performed a bivariate analysis for each of the 35 traits in BJ2014 and SJZ2014, which were the two environments with the most complete datasets (see Methods). In BJ2014, phenotypic and genetic correlations of the 35 traits ranged from 0.06 to 0.63 (median = 0.38) and 0.21 to 0.89 (median = 0.71), respectively (Table 1). In SJZ2014, phenotypic and genetic correlations ranged from 0.06 to 0.64 (median = 0.34) and 0.14 to 0.91 (median = 0.71), respectively.

We further compared genetic correlations within tissues (i.e., EI, FI, and WP) in each environment and found generally higher genetic correlations for agronomic traits than for stalk quality traits in SJZ2014 across all tissues (Figure S3). In BJ2014, however, agronomic and stalk quality traits showed similar genetic correlations between DH and haploid lines, except for agronomic traits measured from WP that were much higher than those from EI or FI.

In BJ2014, narrow-sense heritability (hereafter heritability) of the 35 traits ranged from 0.14 to 0.66 and from 0.20 to 0.61 in the DH and corresponding haploid populations, respectively (Table 1). In SJZ2014, the heritability of the 35 traits ranged from 0.19 to 0.70 and from 0.15 to 0.64 in the DH and haploid populations, respectively. There was no significant difference in heritability between DH and corresponding haploid population for agronomic traits (*p* = 0.13) or for stalk quality traits across environments (*p* = 0.19). However, we observed that agronomic traits generally had higher heritability than stalk quality traits in both environments (Figure S4).

### 2.3. Characterization of Genomic Segment Composition of DH Lines

**Figure 2:**
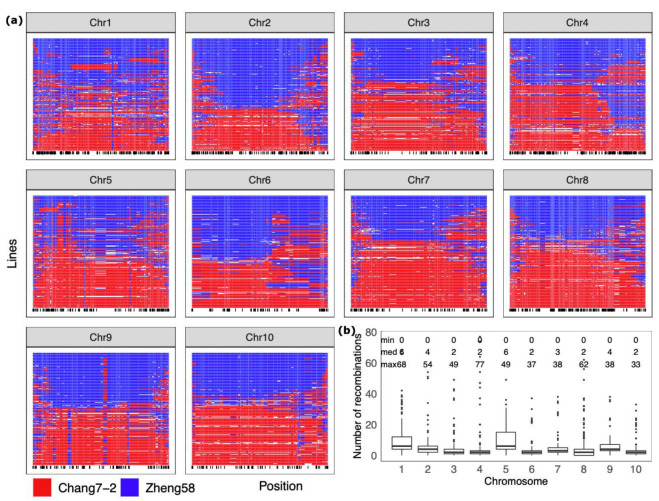
Characterization of genomic segment composition of DH lines: (**a**) reconstructed recombination events in DH lines on each chromosome. The red color marks genotypes of DH lines identical to the parental line Chang7-2, whereas the blue color marks genotypes identical to the other parent, Zheng58. Black bars at the bottom of each chromosome plot indicate the physical positions of SNP markers. (**b**) Number of inferred recombination per chromosome events across all DH lines.

To visualize the genetic composition of DH lines in terms of parental haplotypes, we reconstructed and characterized recombination events on each chromosome of DH lines using the maizeSNP3K chip, which is a subset of the Illumina MaizeSNP50 BeadChip [11]. We found that very long genomic segments with hundreds of millions of base pairs in length or even entire chromosomes were transmitted from parental lines to DH lines (Figure 2a). Median recombination events per chromosome across DH lines ranged from two to four for eight chromosomes; however, chromosomes 1 and 5 showed median value of six recombination events (Figure 2b). Some lines even had zero recombination on some chromosomes. However, maximum number of observed recombination events per chromosome ranged from 33 to 77 across 10 chromosomes, likely in the unknown number of DH lines developed from individuals that experienced larger numbers of generations of recurrent selfing.

### 2.4. Genomic Prediction of Stalk Quality Traits Evaluated in the DH Population

#### 2.4.1. Single-Environment Prediction in the DH Population

**Figure 3:**
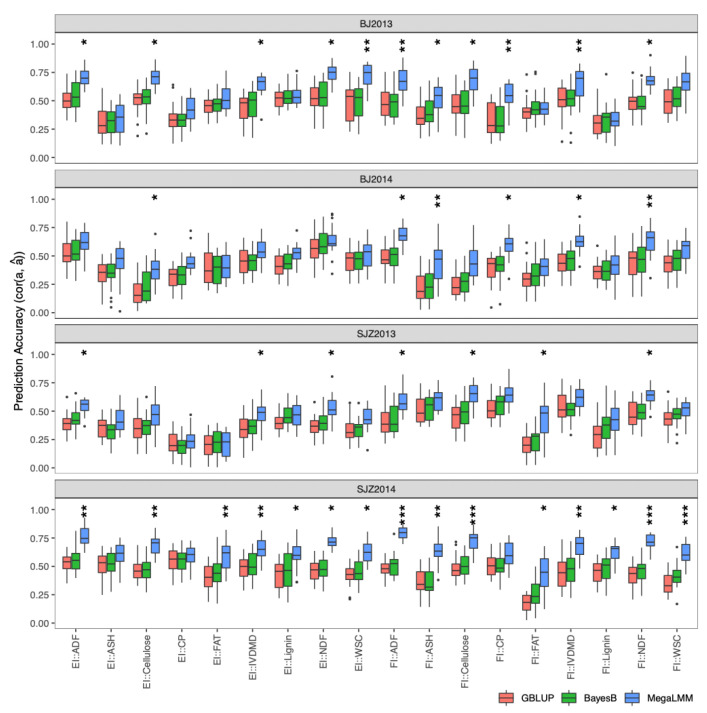
Distribution of prediction accuracies of the 18 stalk quality traits in the DH population across 20 resampling runs of cross-validation. The corrected resampled *t*-test was applied to test differences in prediction accuracy between the GBLUP, BayesB, and MegaLMM (multi-trait) models. Significance levels are indicated above each box, with *** significant at *p* < 0.001, ** significant at *p* < 0.01, and * significant at *p* < 0.05.

To simulate the case in which a breeding program can afford only one location, we performed genomic prediction in each individual environment, leaving out the possible information available from other environments. We compared univariate models taking each trait individually (fitting with GBLUP and BayesB models) to multi-trait models that considered all traits at once (fitting with MegaLMM method). For multi-trait genomic prediction, we considered the “trait-assisted” scenario where the key “focal” traits were measured for a subset of DH lines, but some “secondary” traits were measured for all DH lines. We considered stalk quality traits as focal traits and agronomic traits as secondary traits, because all the agronomic traits in our study can be directly measured in the field, while stalk quality traits require complex chemical experiments in the laboratory or calibration of near-infrared spectroscopy (NIRS) models. We applied CV2-style cross-validation [12] to evaluate model accuracy: all agronomic traits of all DH lines and stalk quality traits of a subset of DH lines were used for model training, and stalk quality traits of the remaining DH lines were predicted and used for model validation. Since the focal and secondary traits were measured on the same plots and therefore shared the same nongenetic sources of variation, the prediction accuracy of each model was estimated as the genetic correlation (cor (a, â)) between observed and predicted genotypic values multiplied by square root of estimated heritability [13], instead of the more traditional Pearson’s correlation (cor(y, â)/sqrt(h^2^)). Estimated prediction accuracies of multi-trait models fit with MegaLMM were significantly higher than both univariates model for 11 (BJ2013), 6 (BJ2014), 7 (SJZ2013), and 15 (SJZ2014) stalk quality traits in the four environments (Figure 3) based on the corrected resampled *t*-test [14]. Between the two univariate models, BayesB showed no significant difference in prediction accuracy compared to GBLUP.

#### 2.4.2. Multi-Environment Prediction in the DH Population

To take advantage of information available from other environments and to simulate the case of multi-environment evaluation of DH lines in breeding practice, we applied multi-trait prediction models to predict each of the stalk quality traits in the DH population. Four different multi-trait linear mixed models (D-D, D-UN, UN-D, and UN-UN) were used in our study. For each model, the uppercase letters before and after the hyphen represent genetic and residual covariance structures, with D = diagonal and UN = unstructured. The diagonal model (D-D) assumes no genetic and residual covariance among locations, and therefore it is equivalent to a single-environment model. In addition, we also fitted the multi-trait model using MegaLMM, which fits a factor-analytic covariance structure for both genetic and residual covariances but uses a novel prior structure for the factor loadings to increase efficiency and power.

**Figure 4:**
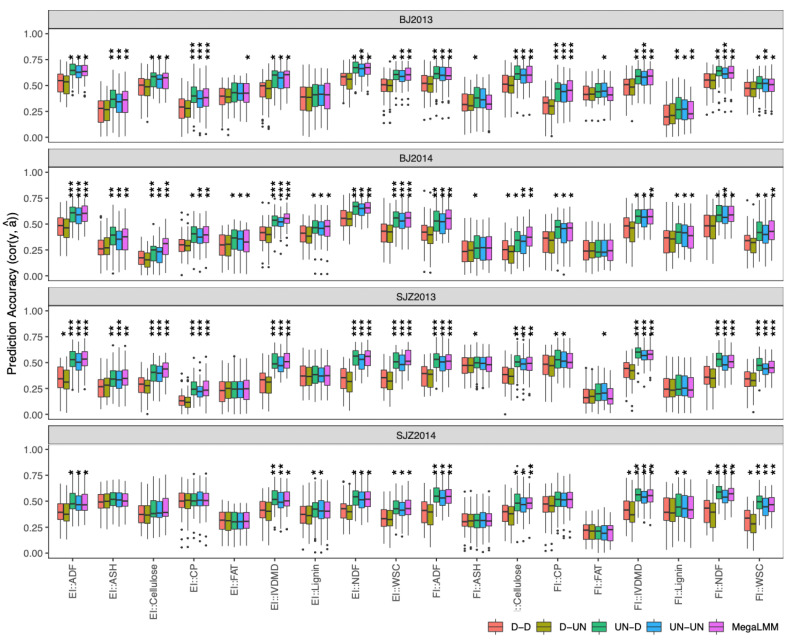
Distribution of prediction accuracies of the 18 stalk quality traits in the DH population across 20 resampling runs estimated by multi-trait models. For each model, the uppercase letters before and after the hyphen represent genetic and residual covariance structures: D = diagonal and UN = unstructured. The corrected resampled *t*-test was applied to test difference in prediction accuracy between the diagonal (D-D) and other multi-trait model, and significance levels are indicated above each box, with *** significant at *p* < 0.001, ** significant at *p* < 0.01, and * significant at *p* < 0.05.

We compared prediction accuracies of different multi-trait models using the prediction accuracy from GBLUP in the single-environment model (D-D) as a baseline. Here, we estimated prediction accuracy as cor (y, â), because the phenotypes of stalk quality traits of the same genotypes were collected from individuals grown in different environments [13]. We found that there was no significant difference between the D-UN and D-D models for all traits in all environments (Figure 4), and almost all other multi-trait models outperformed their counterpart single-environment models (i.e., D-D) in at least three environments for all traits, except for crude fat (EI::FAT and FI::FAT) and lignin (EI::lignin).

#### 2.4.3. Prediction of DH Phenotypes with Both DHs and Haploids in a Single-Environment Trial

Since each DH line is derived from one haploid plant, Geiger et al. [4] suggested that preselection of haploid plants could result in improved per se performance at the DH level. The question that we explored was whether adding the possible information available from haploid lines could improve the prediction accuracy of DH lines compared to using DH data alone. To this aim, we compared prediction accuracies of stalk quality traits of DH lines estimated from agronomic traits of DH lines and all traits of haploid lines (DH + Hap-based analysis) with those estimated from agronomic traits of DH lines only (DH-based analysis) in the context of a single-environment trial.

**Figure 5:**
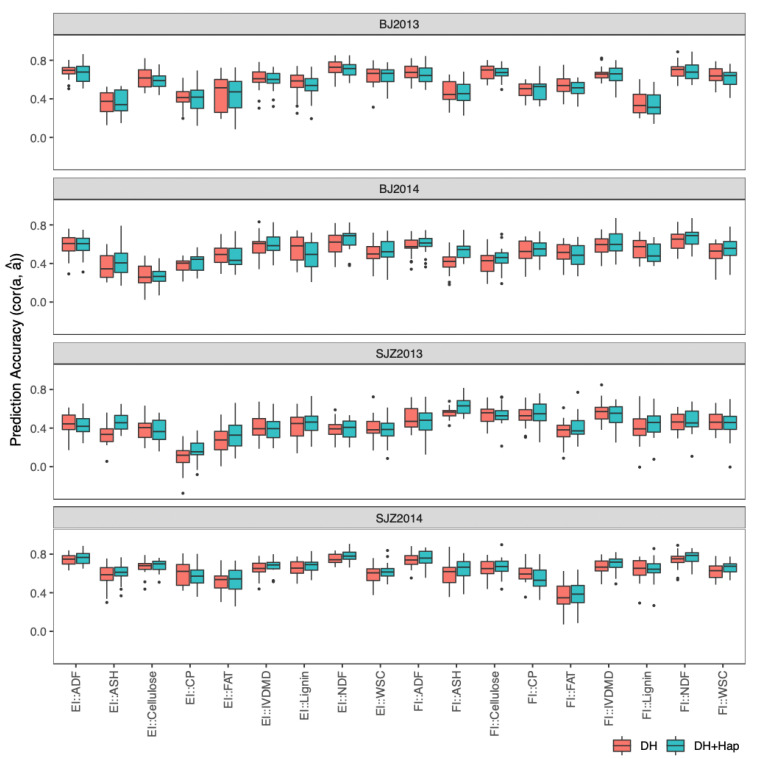
Distribution of prediction accuracies of the 18 stalk quality traits of the DH population across 20 resampling runs estimated by multi-trait models with MegaLMM in single-environment trials. The corrected resampled *t*-test was applied to test difference in prediction accuracy between DH-population-based (DH-based) and DH and haploid populations-based (DH + Hap-based) genomic predictions.

For DH + Hap-based genomic prediction, in each environment, we treated the 18 stalk quality traits of the DH population as focal traits and agronomic traits of DH and all traits of haploid populations as secondary traits, fitted the multi-trait model with MegaLMM, and used the CV2-style cross-validation procedure as described before. We found that no trait showed significant difference in prediction accuracy between DH + Hap-based and DH-based predictions (Figure 5).

#### 2.4.4. Prediction of DH Phenotypes with Both DHs and Haploids in Multi-Environment Trials

**Figure 6:**
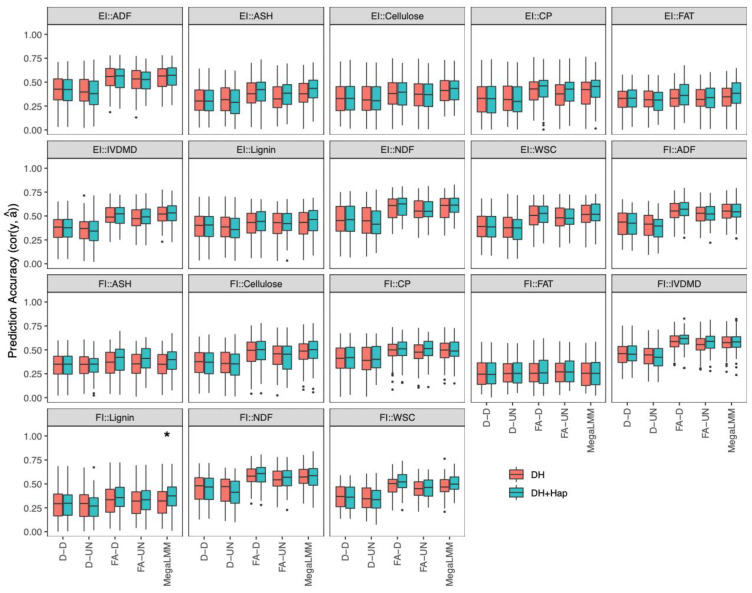
Distribution of prediction accuracies of the 18 stalk quality traits in the DH population across 20 resampling runs estimated by multi-trait models in multi-environment trials. For each multi-trait model, the uppercase letters before and after the hyphen represent genetic and residual covariance structures: D = diagonal, UN = unstructured and FA = factor-analytic. The corrected resampled *t*-test test was applied to test difference in prediction accuracy between DH-population-based (DH-based) and DH and haploid populations-based (DH + Hap-based) genomic predictions across environments. Significance levels are indicated above each box, with * significant at *p* < 0.05.

The availability of phenotypic data of both DH and haploid lines in different environments enabled us to further explore whether DH + Hap-based analysis could improve prediction accuracy of DH lines compared to DH-based analysis in the context of multi-environment trials. In the DH + Hap-based analysis, for predicting each stalk quality trait of DH lines, different environments were treated as different traits and various genetic and residual covariance structures were considered in multi-trait models. Both DH and haploid lines data were used for model training; however, only the DH lines were used for model validation. Since the UN-UN (unstructured genetic and residual covariance structures) model was difficult to converge due to increased numbers of parameters compared to fitting DH data alone, we used the factor-analytic method to model genetic covariance structures (i.e., FA-D and FA-UN). We used cor (y, â) to estimate prediction accuracy since each experiment unit was phenotyped from different individuals, and the prediction accuracy was calculated across environments. We found that only one stalk quality trait (FI::Lignin) showed significantly improved prediction accuracy in the DH + Hap-based analysis compared to the DH-based analysis (Figure 6).

## 3. Discussion

In maize, DH technology can develop pure lines quickly and efficiently from two opposite heterotic pools, resulting in a tremendous number of possible hybrid crosses to be tested in multi-environment field trials (e.g., given 100 DH lines from each of the two opposite heterotic pools, 100 × 100 = 10,000 possible hybrid crosses). This imposes great challenges in field testing with a limited budget and therefore may counterbalance its potential in accelerating hybrid development. As a solution, plant breeders can select their best candidate DH lines beforehand in order to make only a limited number of promising hybrid crosses and evaluate them intensively in multi-environment field trials. In this study, we compared two possible strategies for selecting the best candidate DH lines, namely, phenotypic selection at the haploid level and genomic prediction of unphenotyped DH lines based on whole-genome molecular markers.

Genetic correlations between traits measured in DH and corresponding haploid lines are indicators of achieving selection gain at the DH level from preselection at the haploid level [4]. Geiger et al. [4] estimated genetic correlations between haploid and DH lines in three material sets across four locations and found that estimated genetic correlations of early vigor, silking, plant height, and stover weight per plant ranged between 0.57 and 0.89. In our study, we estimated genetic correlations between haploid and DH lines in two locations (BJ2014 and SJZ2014) separately, and found that the genetic correlations of agronomic traits ranged between 0.57 and 0.89 in BJ2014 (after excluding one possible outlier of 0.21 for EI::RPR) and ranged between 0.57 and 0.91 in SJZ2014 (Table 1). The genetic correlations between haploid and DH lines for agronomic traits were similar to that estimated by Geiger et al. [4], although the stalk quality traits showed lower genetic correlations between DHs and haploids in our study.

In the context of phenotypic selection, the expected selection gain in DH lines from indirect selection of haploid lines can be predicted from the formula $R_{D}=i_{H}r_{A\left(D,H\right)}h_{H}\sigma_{A\left(D\right)}$, where $i_{H}$ is selection intensity in the haploid population, $h_{H}$ is the square root of narrow-sense heritability in the haploid population, $\sigma_{A\left(D\right)}$ is standard deviation of additive effect in the DH population, and $r_{A\left(D,H\right)}$ is additive genetic correlation between DH and haploid populations. The expected selection gain in the DH population with direct selection can be predicted from the formula $R_{D}=i_{D}h_{D}\sigma_{A\left(D\right)}$, where $i_{D}$, $h_{D}$, and $\sigma_{A\left(D\right)}$ are selection intensity, square root of narrow-sense heritability, and standard deviation of additive effect in the DH population, respectively. Therefore, assuming the same selection intensity, indirect selection would be more efficient than direct selection, when the secondary trait (haploid population) is highly heritable and highly correlated to the primary trait (DH population), i.e., $r_{A\left(D,H\right)}h_{H}$ > $h_{D}$ [15]. Since there was no significant difference in heritability between DH and corresponding haploid populations for agronomic traits or for stalk quality traits in our study, indirect selection of haploid lines would be less efficient than direct selection of DH lines using phenotypic evaluations.

Genomic prediction based on whole-genome molecular markers is another strategy for selecting the best DH line candidates without a need to phenotype all DH lines in the field trials. Agronomic traits can be directly measured in the field and showed high heritability. However, stalk quality traits take more effort to measure and showed lower heritability in our study. In the scenario of a single-environment trial with the DH population, we simulated the case where all DH lines are measured for agronomic traits but only a subset of DH lines are measured for stalk quality traits. We found that when integrating the agronomic traits in multi-trait models to predict unphenotyped stalk quality traits, prediction accuracy was significantly increased in comparison with using stalk quality traits alone with traditional univariate GBLUP or BayesB models. Further, in the genomic prediction of multi-environment trials within the DH population, we found that accounting for genetic covariance among locations (i.e., UN-D, UN-UN, and MegaLMM models in Figure 4) could significantly improve prediction accuracy compared to predicting each environment separately (i.e., D-D model); however, considering the residual covariance among locations (i.e., UN-D model) showed no significant difference in prediction accuracy compared to the single-environment prediction. Mathew et al. [16] reported that the UN-D model had similar prediction accuracy to the UN-UN model in presence of strong genotype by environment interaction. Recent genomic prediction studies [17,18,19] in different crop species generally suggested that modeling unstructured genetic covariance (UN) improved prediction accuracy compared to the models with diagonal homogeneous or heterogeneous genetic covariances. Overall, we concluded that considering genetic correlation among traits in single-environment trials as well as genetic covariance among locations can improve genomic prediction accuracy compared to traditional univariate models.

When using agronomic traits assessed from all DH lines to predict stalk quality traits evaluated only in a subset of DH lines, we borrowed the information from DH lines that were phenotyped for agronomic traits but were not phenotyped for stalk quality traits. Further, it is possible to borrow information from corresponding haploid plants/lines to predict unphenotyped DH lines. Since each DH line is derived from a haploid plant, it is reasonable to hypothesize that including the phenotypic information of the haploid plants in genomic prediction could improve prediction accuracy of DH lines compared to using DH lines alone. We used haploid lines created by crossing DH lines with a haploid inducer to test this hypothesis. In the context of single-environment prediction, we added agronomic and stalk quality traits of all haploid lines into the training model to predict stalk quality traits of DH lines that were not phenotyped. In the multi-environment prediction, for predicting stalk quality traits of DH lines, we treated phenotypes of both DH lines and haploid lines in each of the four environments as different traits. However, we found very limited improvement in prediction accuracy between the DH + Hap-based and DH-based predictions in both single-environment and multi-environment analyses (Figure 5 and Figure 6). In addition, in the standard procedure of DH line production, each DH line is derived from a single haploid plant, and therefore plant breeders can only phenotype one haploid plant, rather than haploid lines, to predict corresponding DH lines, which would dramatically decrease the heritability and accuracy of phenotypic evaluation of the haploid population. In summary, preselection of haploid plants shows no benefits in phenotypic selection, and adding haploid information shows very limited merits in genomic prediction of DH lines. Nevertheless, according to Geiger et al. [4], haploid lines can more effectively uncover susceptibility to diseases and environmental constraints compared to corresponding DH lines or testcrosses. Plant breeders may create haploid versions of superior DH lines and evaluate these haploid lines per se in specific stress-prone environments in the final testcross selection stage.

The development of Inbred lines by recurrent selfing for hybrid breeding was first reported by George Shull in 1908, and single-cross hybrids replaced the earlier double-cross varieties in the U.S. in the 1960s [20]. The DH technology based on in vivo haploid induction for line development has become one of the most important tools in maize breeding in the last two to three decades [3]. Although DH lines are considered no different from traditional inbred lines when used for making hybrid crosses, they do have different genomic compositions of founder haplotypes and different homozygosity levels when beginning to be used for testcrossing. Since DH lines are commonly developed from the F_1_ of two parents that have complementary favorable phenotypes, we observed that most of the DH lines had very limited numbers of recombination events on each chromosome, and very large genomic segments with hundreds of millions of base pairs in length or even entire chromosomes were transmitted from parental lines to DH lines (Figure 2). In comparison, inbred lines obtained with six to eight generations of selfing segregating materials have many more recombinations per chromosome and therefore much smaller genomic segments inherited from parental lines. Considering that linkage disequilibrium is an important source of genetic correlation [21], inbred lines developed by selfing may have different landscape of genetic correlations between traits or genetic correlations between lines and testcross hybrids. In hybrid breeding schemes with conventional line development, plant breeders can make testcrosses for selecting best lines as early as in the F_2:3_ generation, and therefore the selected lines are still subject to segregation due to continuous selfing before reaching the desired degree of homozygosity. In contrast, DH lines reach complete homozygosity in one step. The speed and efficiency of DH technology for line development are offset by great numbers of DH lines that are produced without any preselection. We illustrated that preselection of haploid plants is less efficient than direct selection of DH lines using the phenotypic selection. We also proved that taking into account genetic correlation between traits in the single-environment trials or modeling genetic covariance in multi-environment trials can significantly improve genetic prediction. However, integrating additional information of haploid lines does not gain further improvement in accuracy. In the future, more advanced genomic prediction methods that could further take into account the special properties of line development with the DH technology should be developed for DH-line-based hybrid breeding.

## 4. Materials and Methods

### 4.1. Plant Materials and Field Experiments

Two elite maize inbred lines, Zheng58 and Chang7-2, were used as parents for constructing a DH population with approximately 200 lines. Most of the lines of the DH population were developed from the F_1_ generation, but some DH lines were also developed from individuals from higher generations of recurrent selfing. However, the detailed pedigree of these DH lines was not recorded. The DH population was crossed with a haploid inducer line, CAU5 [22], to generate a corresponding haploid population. The field experiments were performed in four environments (i.e., 2 years × 2 locations), namely, in 2013 and 2014 at Shangzhuang experimental station (Beijing, China; denoted BJ2013 and BJ2014) of China Agricultural University, and Shijiazhuang experimental station (Shijiazhuang, China; denoted SJZ2013 and SJZ2014) of Hebei Academy of Agriculture and Forestry Sciences. In each environment, the two populations were planted adjacently to reduce the influence of field heterogeneity, and each population was arranged following a randomized complete block design with two replications. In each block, plants were sown in single rows, 3 m long, with a density of 60,000 plants/ha.

### 4.2. Phenotype Evaluation and Analysis

In each population, three randomly selected plants in each plot were used for phenotypic trait evaluation. A total of 35 phenotypic traits were measured in each field trial, which included 9 stalk quality traits and 5 agronomic traits measured from both the fourth internode above the ground (FI) and the internode under the primary ear (EI), and 7 agronomic traits measured from the whole plant (WP). The 9 stalk quality traits included contents of acid detergent fiber (ADF), ash (ASH), cellulose, crude protein (CP), crude fat (FAT), in vitro dry matter digestibility (IVDMD), lignin, neutral detergent fiber (NDF), and water soluble carbohydrate (WSC). The 5 agronomic traits measured from FI and EI included rind penetrometer resistance measured at the middle of the internode (RPR), fresh weight of the internode (FreshWeight), dry weight of the internode (DryWeight), internode diameter, and internode length. The 7 agronomic traits measured from the WP included dry weight, ear height, fresh weight, leaf angle, leaf length, leaf width, and plant height. Details of phenotyping the stalk quality traits and agronomic traits were described by Hu et al. [23] and Meng et al. [24], respectively.

Within each environment, for the DH population and haploid population separately, a univariate linear mixed was fitted using the sommer package [25]:

$$y_{ij}=\mu +G_{i}+B_{j}+\varepsilon_{ij}$$

where $y_{ij}$ is the plot mean of phenotypic value of genotype i in block j, μ is the overall mean, $G_{i}$ is the random effect of line I, $B_{j}$ is the fixed effect of block j, and $\varepsilon_{ij}$ is the residual. $G_{i}$~N(0, $\sigma_{G}^{2}$), $\varepsilon_{ij}$~N(0, $\sigma_{e}^{2}$). After the model fitting, the random effects of genotypes (i.e., iid. BLUPs) were extracted for downstream analysis.

### 4.3. Genotype Analysis

The DH population was genotyped with the maizeSNP3K chip (3072SNPs), which is a subset of the Illumina MaizeSNP50 BeadChip [11]. The details of genotyping were described by Meng et al. [24]. SNPs were selected using the following criteria: (i) minor allele frequency (MAF) > 5%; (ii) maximum site missing rate < 20%; (iii) maximum site heterozygosity rate < 10%; (iv) maximum individual missing rate < 20%; and (v) maximum rate of individual heterozygous calls < 20%. A total of 1316 markers and 187 lines met these criteria and were used for further analyses.

### 4.4. Estimation of Phenotypic and Genotypic Correlations between Haploid and Doubled Haploid (DH) Populations

We estimated phenotypic and genotypic correlation between haploid and DH populations for each of the 35 traits in BJ2014 and SJZ2014, respectively. The two environments were selected because both populations have very complete phenotypic data. Numbers of available genotypes ranged from 173–177 and 151–159 across traits in the DH and haploid, respectively. We then used medians to substitute missing values for each trait in both populations and ended up with 187 pairs of genotypes for genetic correlation analysis.

We fitted a bivariate linear mixed model with the BGLR package [26]:

(1) $$\left[\begin{array}{c} y_{D}\\ y_{H} \end{array}\right]=\left[\begin{array}{cc} X_{D} & 0\\ 0 & X_{H} \end{array}\right]\left[\begin{array}{c} b_{D}\\ b_{H} \end{array}\right]+\left[\begin{array}{cc} Z_{D} & 0\\ 0 & Z_{H} \end{array}\right]\left[\begin{array}{c} a_{D}\\ a_{H} \end{array}\right]+\left[\begin{array}{c} \varepsilon_{D}\\ \varepsilon_{H} \end{array}\right]$$

where $y_{D}$ and $y_{H}$ are the column vectors of phenotypic data of DH and haploid populations for a trait, and $b_{D}$ and $b_{H}$ are the column vectors of fixed effects, $a_{H}$ are the column vectors of random additive genetic effects, and $\varepsilon_{D}$ and $\varepsilon_{H}$ are the column vectors of residual terms of DH and haploid population, respectively. X_D_/X_H_ and Z_D_/Z_H_ are design matrices relating the fixed and random effects to each genotype. Vectors containing the random effects in Equation (1) are assumed to follow a bivariate normal distribution, centered at zero, and with covariance structure Cov(a, a′) = G_0_⊗A, Cov(ε, ε′) = I⊗R_0_, and Cov(g, ε′) = 0, where G_0_ is a 2 × 2 genetic covariance matrix between DH and haploid lines, ⊗ is the Kronecker product, A is the additive genomic relationship matrix, I is an identity matrix, and R_0_ is a 2 × 2 residual covariance matrix for the DH and haploid populations.

The genotypic correlation was estimated as follows:

(2) $$r_{G\left(D,H\right)}=\frac{\sigma_{G\left(D,H\right)}}{\sqrt{\sigma_{G\left(D\right)}^{2}}\sqrt{\sigma_{G\left(H\right)}^{2}}}$$

For each trait, the phenotypic correlation between the haploid population and the DH population was estimated as the ratio of the phenotypic covariance to the product of the square root of the phenotypic variances for the two populations [27]:

(3) $$r_{P\left(D,H\right)}=\frac{\sigma_{G\left(D,H\right)}+\sigma_{\varepsilon \left(D,H\right)}}{\sqrt{\sigma_{G\left(D\right)}^{2}+\sigma_{\varepsilon \left(D\right)}^{2}}\sqrt{\sigma_{G\left(H\right)}^{2}+\sigma_{\varepsilon \left(H\right)}^{2}}}$$

where $\sigma_{G\left(D,H\right)}$ is genotypic covariance, $\sigma_{\varepsilon \left(D,H\right)}$ is residual environmental covariance, and $\sigma_{G\left(D\right)}^{2}$, $\sigma_{G\left(H\right)}^{2},\;\;\sigma_{\varepsilon \left(D\right)}^{2}$, and $\sigma_{\varepsilon \left(H\right)}^{2}$ are genotypic or environmental variances for traits of DH and haploid, respectively. Since DH lines and haploid lines were planted in different blocks and they were randomly arranged within each block, residual environmental covariance between the DH and haploid population for each trait is zero, i.e., $\sigma_{\varepsilon \left(D,H\right)}$ = 0.

### 4.5. Graphical Genotypes

Based on the filtered genotypic data (1316 markers across 187 lines), SNP markers were sorted on each chromosome according to physical positions, marker genotypes were translated to 0 if identical to Chang7-2 (male parent) and to 1 if identical to Zheng58 (female parent), and a small proportion of heterozygous genotypes were set as NA (missing) because these are likely genotyping errors. After the pretreatments, graphical genotypes of each chromosome were plotted with the geom_rect function implemented in ggplot2 [28], and the box width was proportional to the physical distance between markers. Marker genotypes were colored red if identical to the genotype of Chang7-2 and colored blue if identical to the genotype of Zheng58, and DH lines were sorted according to genotypic similarity to parental lines. Finally, the physical positions of SNP markers were indicated at the bottom of the plot of each chromosome. The number of recombinant events on each chromosome were also estimated by comparing marker genotypes of each DH line and those of parental lines.

### 4.6. Genomic Prediction

#### 4.6.1. Single-Environment Prediction in the DH Population

The objective of single-environment prediction is to simulate the case in which the breeding program can afford field trials in only one environment. Since the phenotypes of the DH population were evaluated in four environments, we analyzed each environment separately, leaving out available information from other environments. To this aim, we calculated additive genomic relationships with the A.mat function implemented in the rrBLUP package [29], and then we fitted GBLUP with rrBLUP and the BayesB model with the BGLR package [26].

To take advantage of available information of all other phenotypes (secondary traits) to predict the phenotype of interest (focal trait), we fitted a multi-trait model using the MegaLMM package [30] and used a CV2-style [12] prediction method where secondary traits of all DH lines were measured but focal traits of some DH lines were not measured. We considered agronomic traits as secondary traits and stalk quality traits as focal traits because all the agronomic traits in our study can be directly measured in the field; however, measurement of the stalk quality traits needs complex chemical experiments in the laboratory or needs near-infrared spectroscopy (NIRS) calibration models to be built based on these chemical values. We simulated the case where all DH lines were measured for agronomic traits but only a subset of DH lines were measured for stalk quality traits.

The prediction accuracy was estimated as $cor\left(a,\hat{a}\right)=c\hat{o}r_{g}\left(\hat{u},y\right)\sqrt{h_{u}^{2}}$, as described by Runcie and Cheng [13], and used a 50:50 training: testing partition for cross-validation for all the three methods. The cross-validation procedure was repeated 20 times with different random partitions. The corrected resampled *t*-test [14] was applied to test mean difference of prediction accuracy between other methods and GBLUP in all genomic prediction analyses.

#### 4.6.2. Multi-Environment Prediction in the DH Population

The DH population was evaluated in four environments for the 35 traits described above. For each of these traits, multi-trait models were fitted, treating phenotypes measured in all environments as different traits. A standard multi-trait linear mixed model was fitted:

(4) y=Xb+Zu+ε

where *y* = (*y*1′, *y*2′, *y*3′, *y*4′)′, *u* = (*u*1′, *u*2′, *u*3′, *u*4′)′, and *ε* = (*ε*1′, *ε*2′, v3′, *ε*4′)′. *y*1, *y*2, *y*3, and *y*4 are the column vectors of phenotypic data in each environment; *u*1, *u*2, *u*3, and *u*4 are the column vectors of random genetic effects in each environment; *ε*1, *ε*2, *ε*3, and *ε*4 are the column vectors of random error terms associated with each environment. X and Z are design matrices relating the fixed and random effects to each genotype. Vectors containing the random effects in Equation (4) are assumed to follow a multivariate normal distribution, centered at zero, and with covariance structure *Cov*(*u*, *u*′) = *G*_0_⊗*K*, *Cov*(*ε*, *ε*′) = *I*⊗*R*_0_, and *Cov*(*g*, *ε*′) = 0, where *G*_0_ is a 4 × 4 genetic covariance matrix, ⊗ is the Kronecker product, *K* is the additive genomic relationship matrix, I is an identity matrix, and *R*_0_ is a 4 × 4 residual covariance for the three locations.

Four different multi-trait linear mixed models (D-D, D-UN, UN-D, and UN-UN) were used in our study, in which G_0_ and *R*_0_ were assumed to have different covariance structures [12]. For each model, the uppercase letters before and after the hyphen represent genetic (*G*_0_) and residual (*R*_0_) covariance structures, where D = diagonal and UN = unstructured. The diagonal model (D-D) assumes no genetic (*G*_0_) and residual (*R*_0_) covariance among locations, and therefore it is equivalent to predict each environment separately. All these multi-trait models were implemented using BGLR with parameters of burnIn = 5000 and nIter = 20,000. In addition, we also fitted the multi-trait model using the MegaLMM package [30].

For all the multi-trait models, we also used the CV2-style cross-validation, which represents a scheme of prediction of lines that have been evaluated in some but not all target environments [12]. To mimic the CV2-style cross-validation, we randomly masked 20% of data in each environment for validation and used the remaining 80% of data for model training. This cross-validation procedure was used to simulate the case in plant breeding where some genotypes have missing phenotypes in some environments but are available in other environments. The cross-validation was repeated 20 times with different random partitions, and the same training and testing data were applied to all the multi-trait models. The prediction accuracy was estimated as *cor*(*y*, *â*), where $\hat{a}$ is the estimated additive genotypic effect.

#### 4.6.3. Use Both DH and Haploid Lines to Predict DH Lines in a Single-Environment Trial

Based on phenotypic data of DH and haploid populations collected in each environment, we explored a scientific question: whether adding the available haploid information could improve prediction accuracy of DH lines compared to using DH lines alone. To answer to this question, we compared prediction accuracies of stalk quality traits of DH lines estimated from agronomic traits of DH lines and all traits of haploid lines (DH + Hap) with those estimated from agronomic traits of DH lines only (DH).

We treated phenotypes of agronomic traits of DH lines and all traits of haploid lines as secondary traits and used 80% of stalk quality traits as focal traits in a multi-trait model for model training, and the remaining 20% of stalk quality traits of DH lines were used for cross-validation. The software MegaLMM 0.1.0. was used for model fitting, and prediction accuracy was calculated in the same way as described above in the single-environment prediction in the DH population.

#### 4.6.4. Use Both DH and Haploid Lines to Predict DH Lines in Multi-Environment Trials

Availability of phenotypes of both DH and haploid lines evaluated in multiple environments allowed us to explore whether using information of both DH and haploid lines could improve prediction accuracy of DH lines compared to using DH lines alone.

In this analysis, for each trait, multi-trait models were fitted treating phenotypes measured from both DH and haploid lines in all environments as different traits for model training, and only masked phenotypic values of 20% DH lines in each environment for cross-validation. To avoid the issues of nonconvergence for solving mixed-model equations, we used the factor-analytic (FA) method to model genetic covariance structures to reduce the numbers of parameters to be estimated. Four regular multi-trait linear mixed models (D-D, D-UN, FA-D, and FA-UN) and MegaLMM were used for model fitting. The prediction accuracy was calculated in the same way as described above in the multi-environment prediction in the DH population.

## Figures and Tables

**Table 1:** Phenotypic ($r_{P}$ ) and genotypic ($r_{G}$ ) correlations between DH and corresponding haploid populations for the 35 traits evaluated from the internode under the primary ear (EI), the fourth internode above the ground (FI), and the whole plant (WP) in BJ2014 and SJZ2014. $h_{D}^{2}$ and $h_{H}^{2}$ represent narrow-sense heritability of DH and haploid populations, respectively.

	BJ2014				SJZ2014			
Trait	$h_{D}^{2}$	$h_{H}^{2}$	$r_{P}$	$r_{G}$	$h_{D}^{2}$	$h_{H}^{2}$	$r_{P}$	$r_{G}$
Agronomic Traits								
EI::DryWeight	0.37	0.32	0.35	0.72	0.39	0.28	0.42	0.72
EI::FreshWeight	0.45	0.38	0.40	0.77	0.53	0.39	0.48	0.82
EI::InternodeDiameter	0.26	0.26	0.31	0.62	0.46	0.37	0.42	0.80
EI::InternodeLength	0.37	0.37	0.38	0.77	0.48	0.47	0.48	0.79
EI::mRPR	0.21	0.20	0.09	0.21	0.56	0.53	0.51	0.88
FI::DryWeight	0.29	0.22	0.24	0.57	0.47	0.42	0.44	0.78
FI::FreshWeight	0.36	0.25	0.34	0.63	0.54	0.45	0.51	0.83
FI::InternodeDiameter	0.25	0.32	0.27	0.62	0.45	0.45	0.41	0.80
FI::InternodeLength	0.54	0.32	0.38	0.73	0.41	0.27	0.32	0.57
FI::mRPR	0.39	0.44	0.40	0.79	0.61	0.44	0.50	0.86
WP::DryWeight	0.36	0.44	0.44	0.78	0.54	0.51	0.59	0.88
WP::EarHeight	0.64	0.47	0.55	0.83	0.52	0.59	0.59	0.83
WP::FreshWeight	0.44	0.41	0.46	0.78	0.62	0.51	0.61	0.89
WP::LeafAngle	0.60	0.61	0.48	0.85	0.70	0.51	0.42	0.76
WP::LeafLength	0.66	0.56	0.63	0.89	0.58	0.64	0.64	0.91
WP::LeafWidth	0.36	0.36	0.46	0.79	0.30	0.41	0.34	0.66
WP::PlantHeight	0.53	0.43	0.52	0.82	0.49	0.57	0.54	0.80
Stalk quality traits								
EI::ADF	0.32	0.32	0.41	0.71	0.37	0.39	0.37	0.64
EI::ASH	0.22	0.25	0.22	0.53	0.33	0.26	0.33	0.59
EI::Cellulose	0.14	0.20	0.14	0.30	0.26	0.27	0.30	0.52
EI::CP	0.31	0.29	0.36	0.72	0.32	0.24	0.28	0.52
EI::FAT	0.21	0.20	0.19	0.43	0.24	0.16	0.15	0.32
EI::IVDMD	0.30	0.27	0.42	0.71	0.34	0.31	0.31	0.53
EI::Lignin	0.26	0.24	0.34	0.59	0.35	0.26	0.23	0.55
EI::NDF	0.41	0.36	0.53	0.79	0.37	0.29	0.34	0.62
EI::WSC	0.29	0.28	0.38	0.67	0.27	0.21	0.27	0.45
FI::ADF	0.39	0.40	0.49	0.81	0.36	0.31	0.29	0.59
FI::ASH	0.17	0.24	0.21	0.46	0.23	0.26	0.19	0.37
FI::Cellulose	0.22	0.30	0.30	0.64	0.29	0.30	0.30	0.53
FI::CP	0.28	0.28	0.27	0.56	0.32	0.28	0.23	0.49
FI::FAT	0.17	0.22	0.06	0.26	0.19	0.15	0.06	0.14
FI::IVDMD	0.39	0.34	0.50	0.80	0.40	0.34	0.29	0.60
FI::Lignin	0.20	0.20	0.25	0.47	0.38	0.29	0.22	0.47
FI::NDF	0.40	0.35	0.51	0.79	0.39	0.29	0.31	0.63
FI::WSC	0.24	0.27	0.34	0.65	0.32	0.28	0.32	0.62

## Data Availability

The data and custom scripts utilized in this paper are documented in the following GitHub repository: https://github.com/hh622/Maize_DH_lines_Multitrait_Prediction (accessed on 18 November 2022).

## Supplementary Materials

The following supporting information can be downloaded at: https://www.mdpi.com/article/10.3390/ijms232314558/s1, Figure S1: Construction of the DH and haploid population; Figure S2: Comparison of coefficients of variation between DH and haploid population for agronomic traits and stalk quality traits; Figure S3: Boxplot of phenotypic and genotypic correlation between DH and haploid populations within tissues for agronomic and stalk quality traits in BJ2014 and SJZ2014; Figure S4: Distributions of narrow-sense heritability of the 35 traits measured in BJ2014 and SJZ2014; Table S1: Means and standard deviations of DH and haploid populations and *t*-test between populations for the 35 traits evaluated in this study.
