# Supplementary material for: Multi-Trait Genomic Prediction Improves Accuracy of Selection among Doubled Haploid Lines in Maize
Source: Int J Mol Sci. 2022 Nov 22;23(23):14558. doi: 10.3390/ijms232314558 (PMC9735914; doi:10.3390/ijms232314558)
Supplement: Supplementary file 1 [file ijms-23-14558-s001.zip › FigureS2 comparison of coefficients of variation between DH and haploid population for agronomic traits and stalk quality traits.pdf]

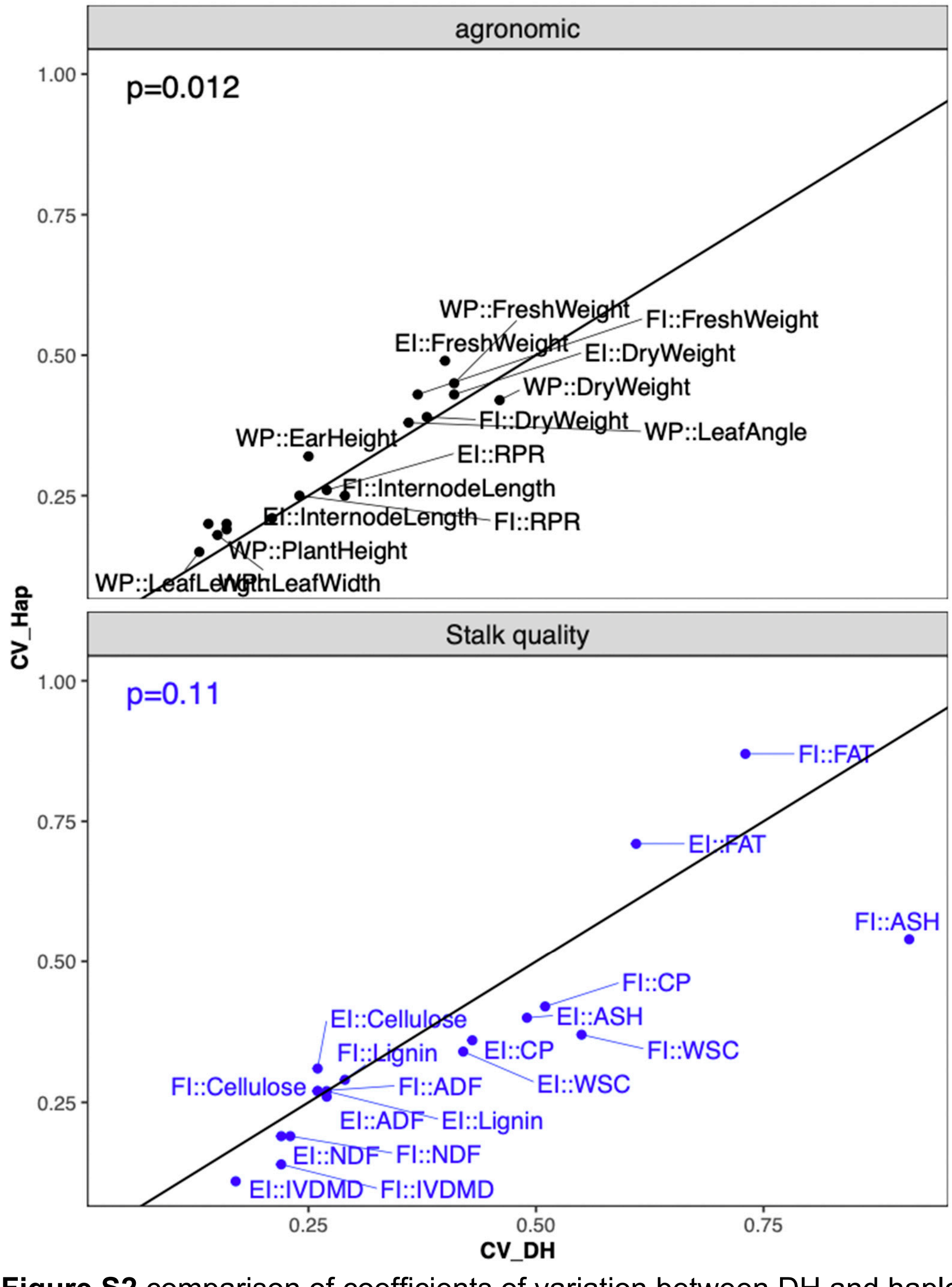

Figure S2 comparison of coefficients of variation between DH and haploid population for agronomic traits and stalk quality traits
