# Supplementary material for: Multi-Trait Genomic Prediction Improves Accuracy of Selection among Doubled Haploid Lines in Maize
Source: Int J Mol Sci. 2022 Nov 22;23(23):14558. doi: 10.3390/ijms232314558 (PMC9735914; doi:10.3390/ijms232314558)
Supplement: Supplementary file 1 [file ijms-23-14558-s001.zip › FigureS3_boxplot_of_phenotypic_and_genotypic_correlation.pdf]

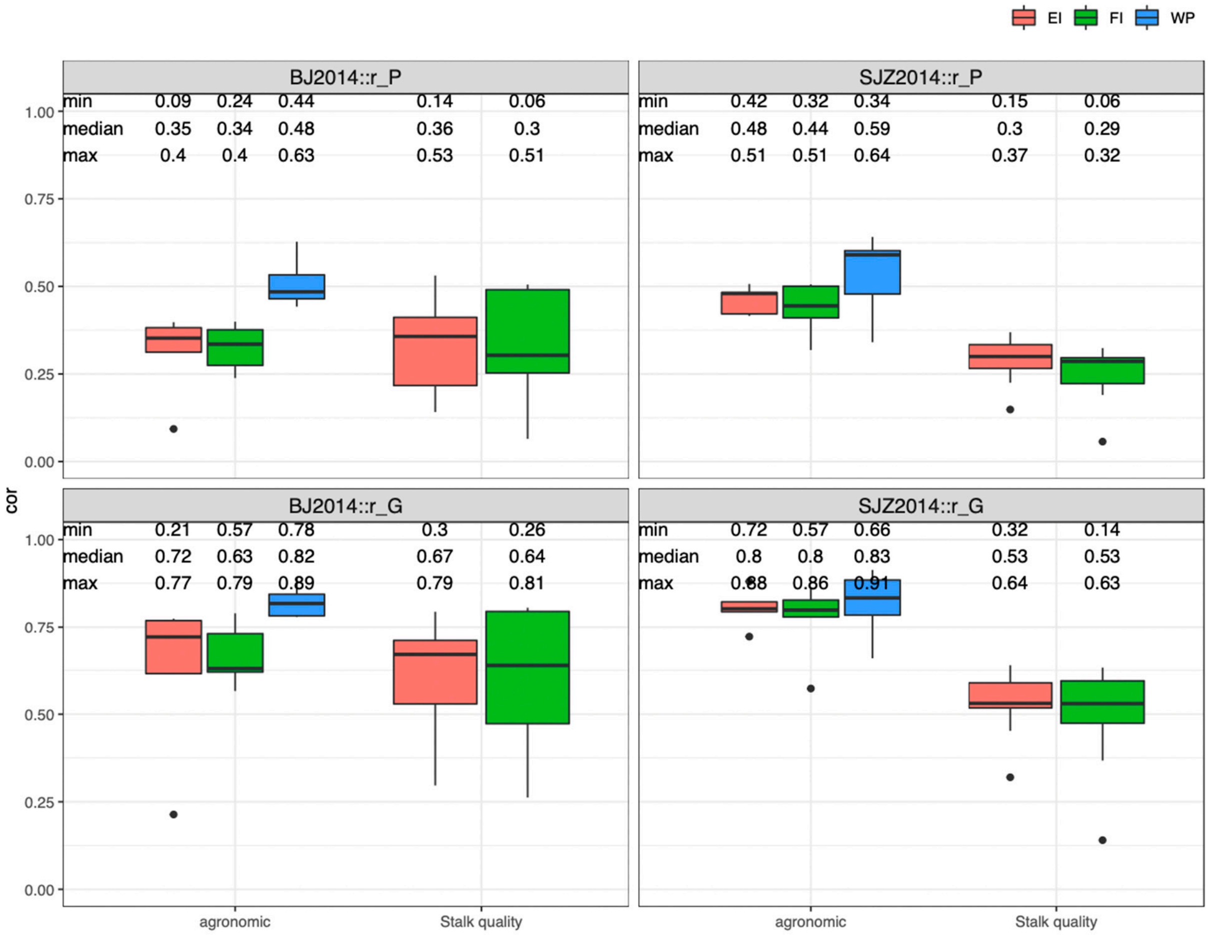

Figure S3 boxplot of phenotypic and genotypic correlation between DH and Haploid populations within tissues for agronomic and stalk quality traits in BJ2014 and SJZ2014.
