# Supplementary figures and images for: Multi-Trait Genomic Prediction Improves Accuracy of Selection among Doubled Haploid Lines in Maize
Source: Int J Mol Sci. 2022 Nov 22;23(23):14558. doi: 10.3390/ijms232314558 (PMC9735914; doi:10.3390/ijms232314558)

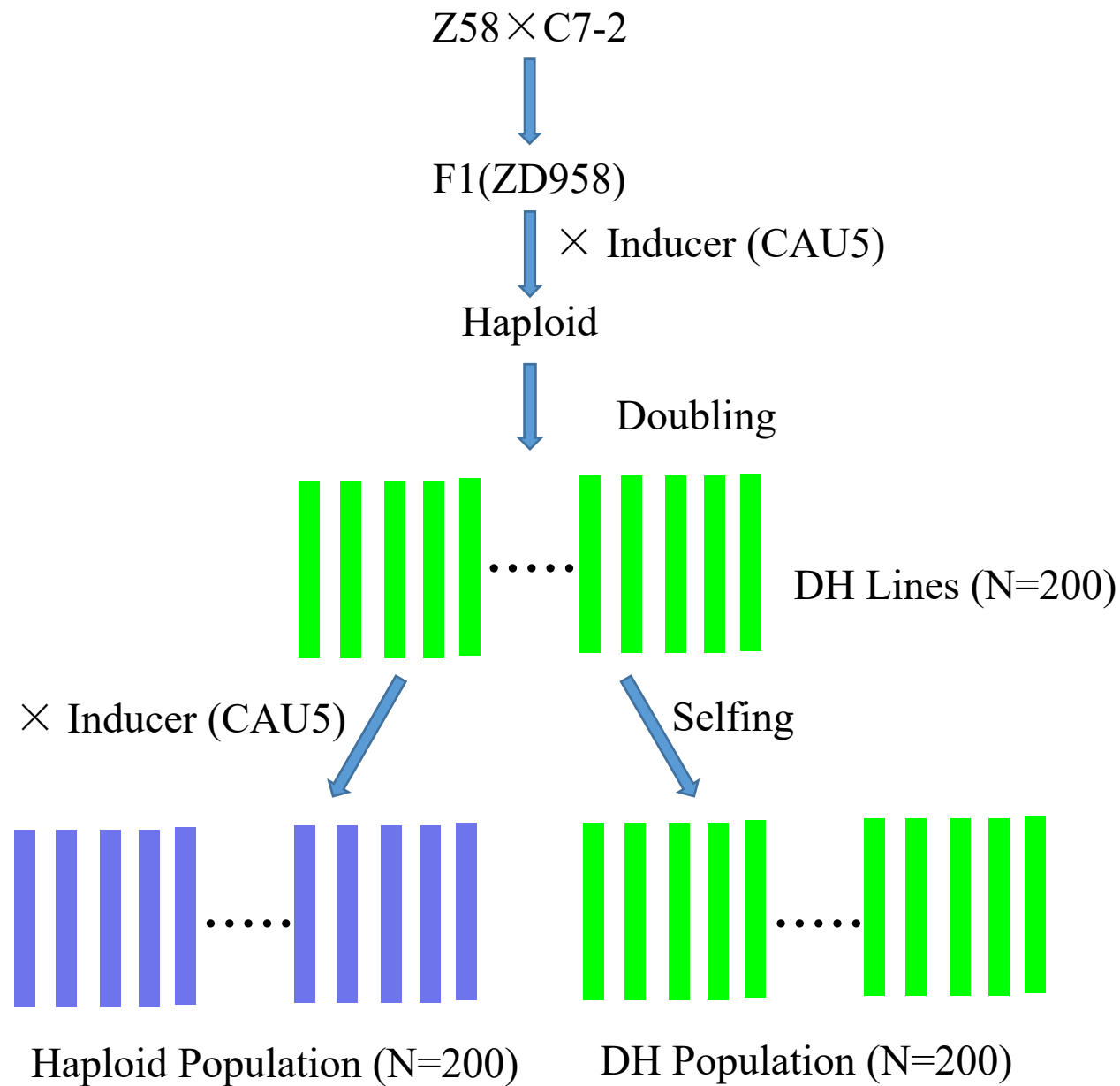

**Figure S1** construction of the DH and haploid population

Supplement: Supplementary file 1 [file ijms-23-14558-s001.zip › FigureS1 construction of the DH and haploid population.pdf]

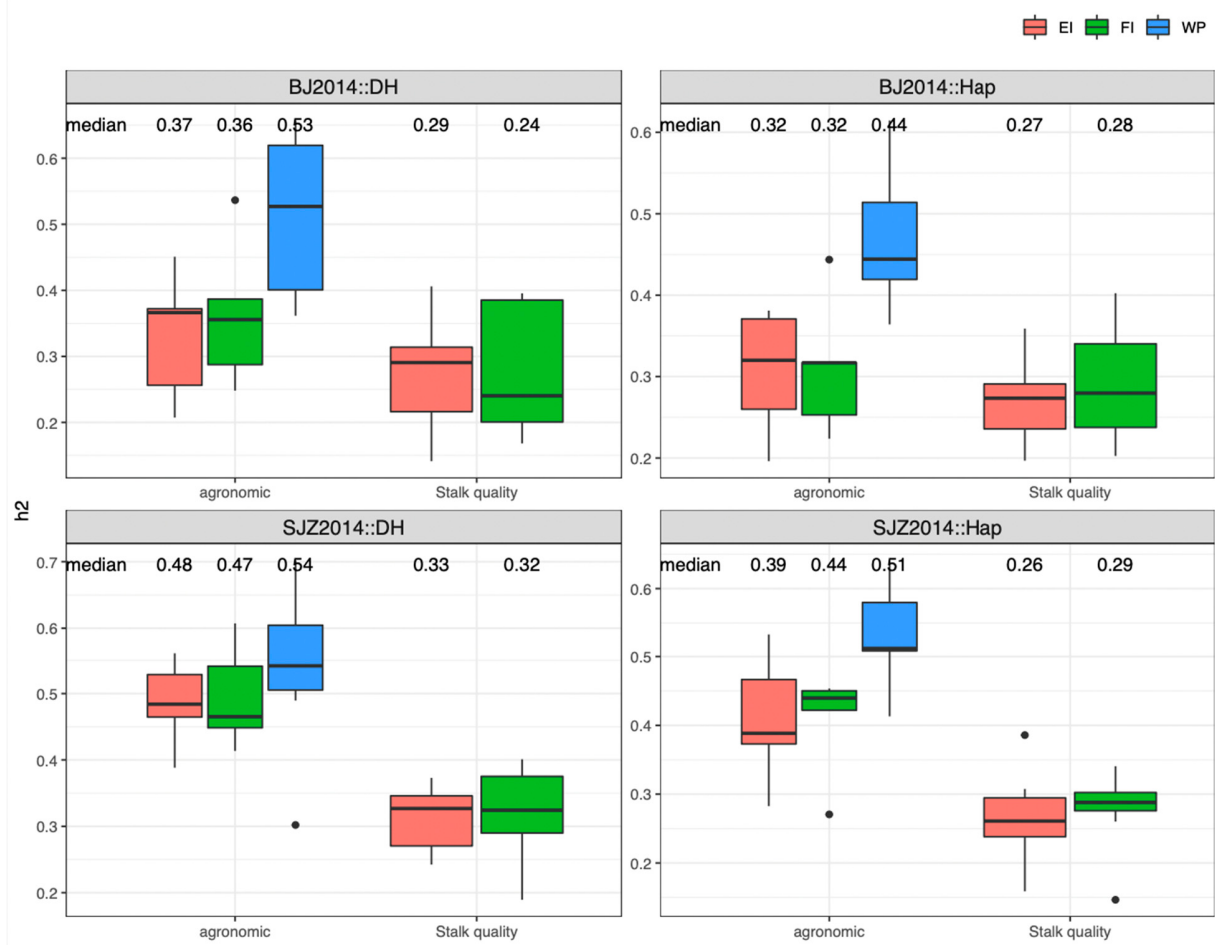

**Figure S4** Distributions of narrow-sense heritability of the 35 traits measured in BJ2014 and SJZ2014.

Supplement: Supplementary file 1 [file ijms-23-14558-s001.zip › FigureS4_Distributions_of_narrow-sense_heritability_of_the_35_traits_measured_in_BJ2014_and_SJZ2014.pdf]
